# microRNAs and the immune response

**DOI:** 10.1016/j.coph.2009.05.003

**Published:** 2009-08

**Authors:** Eleni Tsitsiou, Mark A Lindsay

**Affiliations:** NIHR Respiratory Translational Research Facility, University of Manchester, 2nd Floor Education and Research Centre, Wythenshawe Hospital, Southmoor Road, Manchester M23 9LT, UK

## Abstract

Although the immune response is predominantly controlled at the transcriptional level, microRNA-mediated RNA interference is emerging as an important regulatory mechanism that operates at the translation level. Specifically, recent studies indicate that those miRNAs that are selectively and/or highly expressed in immune cells including the miR-17–92 cluster, miR-150, miR-155, miR-181 and miR-223 have a ‘permissive’ function in the maturation, proliferation and differentiation of myeloid and lymphoid cells. Importantly, these actions of miRNAs often involve interactions with transcription factors. In contrast, the rapid and transient induction of miR-9, miR-146a and miR-155 has been speculated to negatively regulate the acute responses following activation of innate immune through down-regulation of proteins involved in the receptor-induced signalling pathways.

## Introduction

At present, >550 micro (mi)RNAs have been identified in mammalian cells and are known to regulate mRNA translation via the RNA interference pathway. These short RNA sequences of 20–23 nucleotides in length are produced by the processing of full length mRNA-like transcripts [1,2]. miRNA biogenesis involves the initial transcription of primary miRNAs by RNA polymerase II to produce capped, polyadenylated RNA transcripts. In the majority of cases these are subsequently recognised and cleaved within the microprocessor complex that contains the RNase III enzyme Drosha in combination with DGCR8, to create a hairpin RNA of ∼65-nucleotides entitled the precursor microRNA. Precursor miRNAs are then transported into the cytoplasm by exportin 5 and cleaved by the RNase enzyme Dicer to produce the duplex miRNA. The Dicer loaded duplex miRNA is then recruited to argonaute-2 protein by TRBP (the human immunodeficiency virus transactivating response RNA-binding protein) to form the miRNA-induced silencing complex (miRISC) where one strand (often called the passenger strand or * strand) is cleaved to leave the single-stranded mature miRNA (often called the guide strand). At present, miRNAs are believed to either repress mRNA translation or reduce mRNA stability following imperfect binding between the miRNA and the miRNA-recognition elements (MRE) within the 3′ untranslated region (UTR) of target genes. Specificity of the microRNA is thought to be mediated by the ‘seed’ region, that is localised between residues 2–8 at the 5′ end. However, this is influenced by factors such as the presence and cooperation between multiple MREs, the spacing between MREs, proximity to the stop codon, position within the 3′ UTR, AU composition and target mRNA secondary structure [3].

The first indication that miRNAs might regulate the immune responses was a report in 2004 showing selective expression of miR-142a, miR-181a and miR-223 in immune cells [4]. In this study, miR-181a was found to be localised in B-lymphocytes, miR-142a in B-lymphocytes and myeloid cells whilst miR-223 was confined to myeloid cells. Since this initial observation, miRNAs have been implicated in the regulation of maturation, proliferation, differentiation and activation of immune cells. In this review, we briefly describe the recent studies that have examined the function of miRNAs in the innate and acquired immune response [5–7].

## Innate immune response

**Figure 1:**
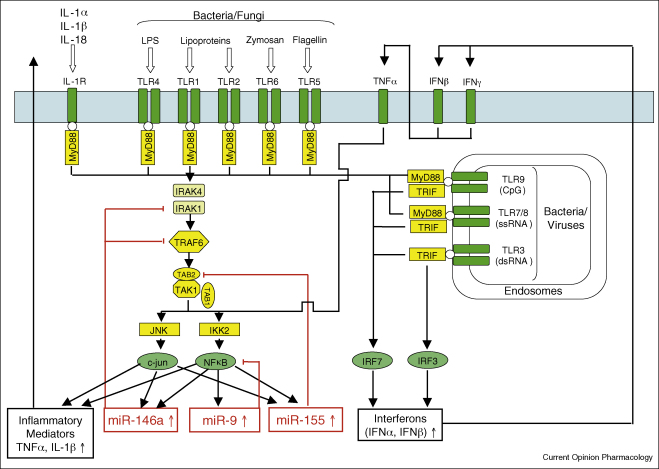
Overview of the role of miR-9, miR-146a and miR-155 during activation of the innate immune responses. The illustration summarises the mechanisms that regulate the induction and action of miR-9, miR-146a and miR-155 following activation of the Toll-like/interleukin-1 receptor superfamily. Abbreviations: IL, interleukin; IFN, interferon; IRAK, IL-1 receptor activated kinase; IRF, interferon regulatory factor; JNK, c-jun N-terminal kinase; NF, nuclear factor; TAB, TAK-1 binding protein; TAK, Toll activated kinase, TLR, Toll-like receptor; TNF, tumour necrosis factor; TRAF, TNF receptor associated factor.

The innate immune response provides the initial defence against infection by external pathogens and is predominantly mediated via myeloid cells such as macrophages, dendritic cells, monocytes and neutrophils. The presence of pathogens is commonly detected by tissue macrophages and dendritic cells via families of pattern recognition receptors that bind conserved molecules within bacteria, fungi and viruses. Many families of pattern recognition receptors have been identified although the best characterised are the Toll-like and interleukin-1 receptors (TIRs). As the name implies, the TIRs can be subdivided into the Toll-like receptors (TLR) that are composed of 11 members (named TLR-1 to TLR-11) and the interleukin (IL)-1 receptors that have 10 members (Figure 1).

### miR-9, miR-146a and miR-155 regulate the activation of myeloid cells

Recent investigations in macrophages, monocytes, neutrophils, epithelial cells and in whole animal studies have shown that the activation of TIRs and TNFα receptor results in rapid expression of a host of miRNAs including let-7, miR-9, miR-99b, let-7e, miR-125a, miR-132, miR-146a, miR-146b, miR-155, miR-187 and miR-223 [8^••^,9^•^,10–13] (Figure 1). Of these miRNAs, only miR-146a and miR-155 appear to be induced in multiple cell types. Studies into the mechanism of LPS-induced miR-9, miR-146a and miR-155 expression demonstrate a central role of the pro-inflammatory transcription factor nuclear factor (NF)-κB [8^••^,11,13]. However, signalling through the c-jun-N-terminal kinase (JNK) also appears to regulate poly I:C (TLR-3) and TNFα-induced miR-155 expression in murine macrophages [9^•^]. Examination of the targets of these miRNAs has shown them to down-regulate proteins involved in the TIR signalling pathway including nuclear factor-κB1 (NF-κB1) (miR-9), tumour receptor factor associated factor-6 (TRAF6) (miR-146a), IL-1 receptor activated kinase-1 (IRAK1) (miR-146a), TAB2 (miR-155), Fas-associated death domain protein (miR-155), IκB kinase ɛ (IKKɛ) (miR-155) and receptor interacting serine–threonine kinase 1 (Ripk1) (miR-155) [8^••^,9^•^,11,13]. This observation has led to the speculation that miR-9, miR-146a and miR-155 might negatively regulate TIR-induced responses associated with the innate immune response although there is presently little experimental evidence to support that contention. Interestingly, our own studies examining the effect of inhibitors and mimics of miR-146a upon the IL-1β-induced in alveolar A549 epithelial cells have shown that miR-146a negatively regulates chemokine release but not through down-regulation of TRAF6, IRAK1 or other protein involved in the IL-1β signalling pathway [14].

### miR-155, miR-223 and the miR-17–19 cluster regulate myeloid proliferation and differentiation

In addition to their role in the acute response to pathogens, miR-155, miR-223 and the miR-17–92 cluster have also been implicated in myeloid proliferation and differentiation. A role for miR-155 was first suggested from the examination of bone marrow myeloid cells obtained from LPS exposed mice [15]. Additional support for a role in proliferation was obtained from the studies of enforced miR-155 expression in haematopoietic stem cells that produced a myoproliferative disorder similar to acute myeloid leukaemia (AML). Microarray analysis indicated that the actions of miR-155 were mediated through down-regulation of transcription factor known to regulate haematopoietic development including Cutl1, Arntl, Picalm, Jarid2, Csf1r, HIF1α, Cebpβ, Bach1 and PU.1 (see later) [15]. Studies in human cord blood CD34^+^ haematopoietic progenitor cells induced to differentiate into monocytes upon exposure to macrophage-colony stimulating factor (M-CSF) also implicated the miR-17–92 cluster [16]. Under these circumstances, the reduction in miR-17–92 expression led to an increase in the transcription factor acute myeloid leukaemia-1 (AML1), the DNA-binding subunit of the haematopoietic transcription factor core-binding factor (CBF), which caused expression of genes involved in myeloid cell differentiation [16].

In contrast to miR-155 and the miR-17–92 cluster, miR-223 negatively regulates the proliferation and differentiation of neutrophils through down-regulation of the transcription factor Mef2c, a known promoter of myeloid progenitor proliferation. This conclusion is based upon observations in a miR-223 knockout mouse, which demonstrated increased neutrophil numbers, spontaneous inflammation in the lung and exaggerated tissue destruction following LPS challenge [17]. Interestingly, it appears that miR-223 is selectively expressed in neutrophils and macrophages but not monocytes and lymphocytes and this process is regulated by competitive binding between transcription factors. Thus, increased miR-223 expression results from the displacement of the inhibitor NFI-A by two stimulatory transcription factors, C/EBPa and PU.1 [18,19]. Significantly, this process is driven by the miR-223-mediated down-regulation in NFI-A protein expression [18,19].

## Acquired immune response

**Figure 2:**
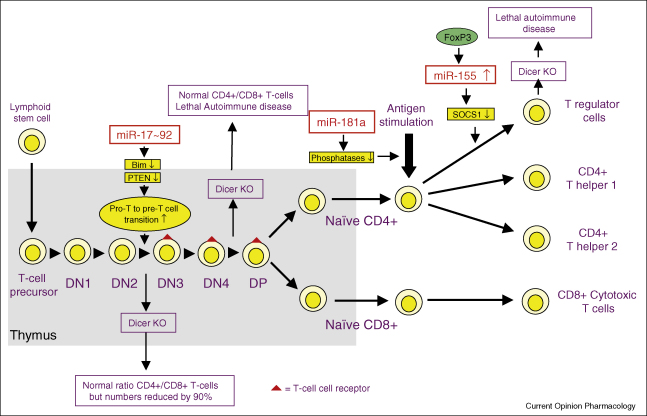
Overview of the role of miRNAs during the maturation, activation and differentiation of T-lymphocytes. The illustration summarises the role of miRNAs in the regulation of T-lymphocytes. Because nothing is known of the role of miRNAs in the development of memory T-cells and other T-cells subtypes, these have been omitted. Abbreviations: CD, cluster designation; DN, double negative; DP, double positive; PTEN, phosphatase and tensin homologue.

**Figure 3:**
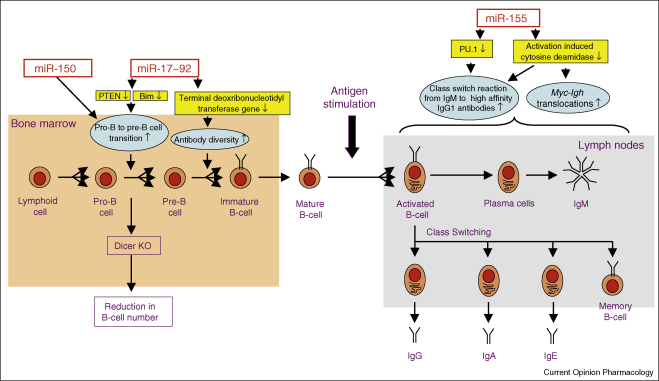
Overview of the role of miRNAs during the maturation, activation and the differentiation of B-lymphocytes. The illustration summarises the role of miRNAs in the regulation of B-lymphocytes. Abbreviations: Ig, antibody; PTEN, phosphatase and tensin homologue.

The acquired immune response involves the selective recognition and removal of invading pathogens by the T-cell receptors and antibodies expressed by T-lymphocytes and B-lymphocytes, respectively. The maturation (production of TCR and antibodies), proliferation, differentiation and activation of lymphocytes involve multiple compartments and a complex and tightly controlled pathway (Figures 2 and 3). This has meant that the majority of the studies looking into the role of miRNAs in the acquired immune response have been performed in knockout and transgenic mice.

### DICER knockout mice demonstrates a role of miRNAs in T-cells

Initial studies into the role of miRNAs in the acquired immune response involved the knockout of DICER, an RNase III enzyme, that is crucial to the production of mature miRNAs (Figure 2). Dicer knockout during early development (prior to DN3) showed that miRNAs were not required for CD4^+^ and CD8^+^ T-cells lineage commitment but were crucial for T-cell proliferation that was reflected in the 90% reduction in circulating cells [20^•^]. At least part of the action of DICER knockout appears to be mediated through loss of the miR-17–92 miRNA cluster that targets down-regulation of the pro-apoptotic protein, Bim and the tumour suppressor, PTEN [21]. Thus, conditional over-expression of this cluster of seven miRNAs (miR-17-5p, miR-17-3p, miR-18a, miR-19a, miR-20a, miR-19b and miR-92) at the DN1 stage resulted in auto-immunity that was characterised by enhanced T-cell proliferation and survival, particular effector CD4^+^ T-cells. Dicer knockout at the later stages of T-cell maturation (DN/DP transition) showed that miRNAs were required for the generation of CD4^+^ Treg cells and Th2 phenotype [22,23]. The importance of miRNAs in FoxP3^+^ CD4^+^ Treg development and immunosuppressive activity has been confirmed by FoxP3 driven deletion of DICER which results in mice that die of spontaneous inflammation and autoimmune disease [24–26].

### miR-155 and miR-181a regulate T-cell response

Although the DICER knockout studies have indicated that miRNAs are important in maintaining the T-cell driven immune response, with the exception of miR-155 and miR-181a much less is known regarding the role of individual miRNAs. Observations in miR-155 knockout animals have shown that these animals are healthy but are unable to mount an acquired immune response [27^•^,28^•^]. The role of miR-155 is still uncertain although it would appear that miR-155 is important in both the T-cell and B-cell-mediated response. Examination of the profile of T-cell expression in miR-155 knockout mice show intrinsic bias towards Th2 phenotype which results from increased expression of the Th2 cytokines and the transcription factor, c-maf [27^•^,28^•^]. Interestingly, recent studies have shown that the transcriptional factor FoxP3, that is required for differentiation into CD4^+^ Treg cells, also induced miR-155 expression. Subsequent analysis using miR-155 knockout animals has established that miR-155 is required for the differentiation and proliferation but not the immunosuppressive action of CD4^+^ Treg cells [29,30]. Mechanistic studies indicate that miR-155 down-regulates the protein suppressor of cytokine signalling 1 (SOCS1) which promotes competitive fitness and increased proliferation through the IL-2 and STAT5 signalling pathways [29] (Figure 2).

The original report by Chen *et al.* showing that miRNAs are differentially expressed in immune cells also showed that over-expression of miR-181a is associated with a reduction in CD8^+^ T-cells [4]. A recent report has shown that increased expression of miR-181a but not miR-181c is involved in the production of CD4^+^ and CD8^+^ double-positive (DP) T-cells [31]. Intriguingly, the differential actions of miR-181a and miR-181c resulted from differences in the stem loop of the pre-miRNAs and not from the one nucleotide change in the guide sequences of miRNAs [31].

Both miR-155 and miR-181a have also been implicated in the regulation of acute response following activation of CD4^+^ T-cells. Comparison of the response in CD4^+^ T-cells obtained from normal and knockout mice indicates that miR-155 was required for the release of cytokines such as IL-2 and IFNγ [27^•^]. Similarly, the changes in the expression of miR-181a, which are increased during T-cell maturation (DN1–3) before dropping in CD4^+^ and CD8^+^ T-cells, have also been shown to modulate TCR signalling [32^••^]. In this case, elevated miR-181a levels increased the strength and lowered the threshold required for TCR signalling in CD4^+^ T-cells through coordinated down-regulation of the tyrosine phosphatases, Src homology 2 domain-containing protein-tyrosine phosphatase (SHP)-2 and protein-tyrosine phosphatase (PTP)-N22, and the ERK-specific, dual specificity phosphatases (DUSP)-5 and DUSP-6 [32^••^].

### DICER knockout mice demonstrate a role of miRNAs in B-cell function

The ablation of DICER in early B-cells maturation was shown to block the pre-B-cell to pro-B-cell transition resulting in a reduction in the number of B-cells [33] (Figure 3). As with T-cells, this blockage appeared to be partly mediated through reduction in the expression of the miR-17–92 cluster which results in the increased expression of its targets, the pro-apoptotic protein Bim and the tumour suppressor, PTEN [21,33,34]. Over-expression of miR-150 which is normally increased during the maturation of both T-cell and B-cell has also been shown to block the pro-B-cell to pre-B-cell transition although this had no effect upon the formation of either CD4^+^ and CD8^+^ T-cells or myeloid cells [35]. The examination of antibody production in DICER knockout animals showed intact Ig gene rearrangements in immunoglobulin heavy (IgH) and kappa chain loci, but increased sterile transcription and usage of D(H) elements of the DSP family in IgH, and increased N sequence addition in Igκ because of deregulated transcription of the terminal deoxynucleotidyl transferase gene [33].

### miR-155 regulates the B-cell response

The availability of knockout and transgenic mouse models has also established the importance of miR-155 in B-cell-mediated antibody production and immunity [27^•^,28^•^]. Although these animals have normal circulating B-cell numbers, they were found to be immuno-deficient, had reduced numbers of germinal centre B-cells and failed to develop an immune response to pathogens following immunisation [27^•^,28^•^]. These defective responses appear to be mediated through the involvement of miR-155 in B-cell production of high affinity IgG1 antibodies, the development of B-cell memory and the production of cytokines such as TNFα and lymphotoxin-α/β [28^•^,36]. Mechanistic studies have shown that the reduction of IgG1 switching is partly mediated through increased expression of the transcription factor PU.1, which has been identified as a direct target of miR-155 [36] (Figure 3). Recent studies have also identified the activation-induced cytidine deaminase (AID) as a target of miR-155 [37^•^,38^•^]. This enzyme catalyses the deamination of cytosine residues and introduction of U:G mismatches, that is required for the generation of the secondary antibody repertoire through somatic hypermutation, gene conversion and class switching from IgM to IgG1 [37^•^,38^•^]. Examination of the role of miR-155 targeting of AID through disruption of a miR-155 binding site in its 3′ UTR resulted in elevated levels of AID mRNA and protein, an increase in class switching of IgM to high affinity IgG1, elevated *Myc*–*Igh* translocations but had no effect upon somatic hypermutation [37^•^,38^•^]. Studies in isolated mouse spleen B-cells have shown that activation via BCR or LPS increased miR-155 expression via an NFAT/calcinerin and NF-κB dependent pathway, respectively [28^•^] (Figure 3).

## Conclusion

This review has outlined the recent evidence demonstrating the importance of miRNAs in the maturation, proliferation, differentiation and activation of the immune response. Significantly, these reports suggest that miRNA-mediated RNA interference regulates the immune response through the selective expression of a range of miRNAs including miR-146a, miR-155, miR-181a and miR-223. However, in order to appreciate the role of miRNAs in the immune system, it is important to place this in the context of the complex and coordinated changes at the transcription level. Thus, unlike transcription factors that generally drive protein production, miRNAs are thought to inhibit mRNA expression and thereby eliminate unwanted proteins. Given this observation, miRNAs could be described as having a ‘permissive’ function during the maturation, proliferation and differentiation of the immune cells. As an example, studies in DICER knockout animals have indicated that expression of the miR-17–92 cluster ‘permits’ the proliferation of T-cell and B-cell through down-regulation of the pro-apoptotic proteins, Bim and the tumour suppressor, PTEN [21,33,34]. Interestingly, when the expression of these miRNAs changes during development these act like rheostats to control immune responses. Thus, the changing expression of miR-181a expression during T-cell maturation has been shown to control the threshold and strength of signalling during antigen–TCR interaction through down-regulation of multiple protein phosphatases [32^••^].

As might be expected from the changing transcriptional profile, another important observation from recent studies is that the targets and function of individual miRNAs appear to be dependent upon cellular context, that is miRNAs can only target mRNA that has been transcribed. Thus, studies of miR-155 in transgenic and knockout mice have shown that miR-155 regulated the development of Treg through down-regulation of SOCS1 [29,30], B-cells antibody class switching through down-regulation of AICD [37^•^,38^•^] and myeloid proliferation and differentiation via down-regulation of multiple transcription factors [15].

In contrast to the maturation, proliferation and differentiation phases, it has been speculated that the rapid (and reversible) changes in miRNA expression observed during activation of the innate and acquired immune response are involved in negative and positive regulation of the associated inflammatory responses. Thus, examination of the TIR-induced response has shown increased miR-9, miR-146a and miR-155 expression and subsequent down-regulation of proteins involved in the TIR signalling pathway [8^••^,9^•^,10–13]. Unfortunately, these reports have not examined the functional consequences either of the changes in miRNA expression and whether this is linked to down-regulation of the TIR-signalling pathway. In addition, studies will also need to determine the relationship between miRNAs and multiple negative feedback pathways that are known to regulate TIR-induced responses including the release of anti-inflammatory mediators. Of note, a recent report has suggested that increased miR-106a expression in activated Jurkat T-cells is responsible for the down-regulation of the anti-inflammatory cytokine, IL-10 [39].

## References and recommended reading

Papers of particular interest, published within the period of review, have been highlighted as:

• of special interest
•• of outstanding interest
